# Binding Affinity Characterization of Four Antennae-Enriched Odorant-Binding Proteins From *Harmonia axyridis* (Coleoptera: Coccinellidae)

**DOI:** 10.3389/fphys.2022.829766

**Published:** 2022-03-08

**Authors:** Cheng Qu, Zhao-kai Yang, Su Wang, Hai-peng Zhao, Feng-qi Li, Xin-ling Yang, Chen Luo

**Affiliations:** ^1^Institute of Plant Protection, Beijing Academy of Agriculture and Forestry Sciences, Beijing, China; ^2^Department of Applied Chemistry, Innovation Center of Pesticide Research, China Agricultural University, Beijing, China; ^3^College of Plant Protection, Shandong Agricultural University, Taian, China

**Keywords:** *Harmonia axyridis*, odorant-binding proteins, fluorescence competitive binding assays, molecular docking, volatile compounds

## Abstract

*Harmonia axyridis* is an important natural enemy that consumes many agricultural and forestry pests. It relies on a sensitive olfactory system to find prey and mates. Odorant-binding proteins (OBPs) as the first-step of recognizing volatiles, transport odors through sensillum lymph to odorant receptors (ORs). However, little is known about the molecular mechanisms of *H. axyridis* olfaction. In this study, four *H. axyridis* antenna specific OBP genes, *HaxyOBP3*, *5*, *12*, and *15*, were bacterially expressed and the binding features of the four recombinant proteins to 40 substances were investigated using fluorescence competitive binding assays. Three-dimensional structure modeling and molecular docking analysis predicted the binding sites between HaxyOBPs and candidate volatiles. Developmental expression analyses showed that the four HaxyOBP genes displayed a variety of expression patterns at different development stages. The expression levels of *HaxyOBP3* and *HaxyOBP15* were higher in the adult stage than in the other developmental stages, and *HaxyOBP15* was significantly transcriptionally enriched in adult stage. Ligand-binding analysis demonstrated that HaxyOBP3 and HaxyOBP12 only combined with two compounds, β-ionone and p-anisaldehyde. HaxyOBP5 protein displayed binding affinities with methyl salicylate, β-ionone, and p-anisaldehyde (K_i_ = 18.15, 11.71, and 13.45 μM). HaxyOBP15 protein had a broad binding profile with (E)-β-farnesene, β-ionone, α-ionone, geranyl acetate, nonyl aldehyde, dihydro-β-ionone, and linalyl acetate (K_i_ = 4.33–31.01 μM), and hydrophobic interactions played a key role in the binding of HaxyOBP15 to these substances according to molecular docking. Taken together, HaxyOBP15 exhibited a broader ligand-binding spectrum and a higher expression in adult stage than HaxyOBP3, 5, and 12, indicating HaxyOBP15 may play a greater role in binding volatiles than other three HaxyOBPs. The results will increase our understanding of the molecular mechanism of *H. axyridis* olfaction and may also result in new management strategies (attractants/repellents) that increase the biological control efficacy of *H. axyridis*.

## Introduction

Insects rely on sensitive olfactory systems to perceive chemical signals from the environment, which are important in locating mates, detecting food sources, and finding suitable oviposition sites (Sato et al., 2008; Brito et al., 2016). The interaction between odorant-binding proteins (OBPs) and odorants is the first-step to recognize chemicals, transporting external odors through sensillum lymph to odorant receptors (ORs; Laughlin et al., 2008; Glaser et al., 2015; Elfekih et al., 2016; Pelosi et al., 2018). In *Antheraea polyphemus*, the first OBP was identified showing the function of sex pheromone binding (Vogt and Riddiford, 1981). Since then, many OBPs have been identified in species from different insect orders, including Lepidoptera (Zhu et al., 2013; Yang et al., 2017; Zhang et al., 2017b), Diptera (Zhao et al., 2018; Chen et al., 2019), Hemiptera (Wang et al., 2017; Sun et al., 2020), Neuroptera (Li et al., 2015), and Coleoptera (Bin et al., 2017; Liu et al., 2018).

It is helpful for identifying the function to study the OBPs expression patterns (Gong et al., 2014; Tang et al., 2019). OBPs have a variety of functions depending on their distribution (Sun et al., 2012; Xue et al., 2016; Li et al., 2019b). Antennae-specific OBPs play important roles in detecting sex pheromones and plant volatiles (Sun et al., 2014; Liu et al., 2020).

The Asian multicolored ladybird beetle, *H. axyridis* (Coleoptera: Coccinellidae), as an important natural enemy, can prey on many pests, including aphids, whiteflies, and thrips. Since the early 21 century, this species has been successfully used to control pests of crops (Koch, 2003; Pervez and Omkar, 2006; Wang et al., 2015). *Harmonia axyridis* is an effective biological control agent, but it can also be a pest in some situations (Soares et al., 2007; Koch and Costamagna, 2016; Ovchinnikov et al., 2019). It may compete with native predators for common food resources, and bring pollution to wine production (Pickering et al., 2004; Katsanis et al., 2013; Grez et al., 2016).

Predators used aphid alarm pheromones and pest-induced volatiles to locate pest (Al Abassi et al., 2000; Hatt et al., 2019), which is an important communication way of pest-crop-natural enemy interactions in agricultural fields. It is necessary for enhancing natural enemies’ biological control efficacy to understand their olfactory systems. Therefore, the interaction of *H. axyridis* with plant volatiles and aphid pheromones may be important for enhancing the effectiveness of *H. axyridis* as a biological control agent.

We previously identified 19 putative OBPs and characterized their tissue expression patterns by quantitative real-time PCR (qRT-PCR) based on antennae and whole-body transcriptomes of *H. axyridis* (Qu et al., 2021). *HaxyOBP3* (NCBI accession number MT150141), *HaxyOBP5* (NCBI accession number MT150143), *HaxyOBP12* (NCBI accession number MT150150), and *HaxyOBP15* (NCBI accession number MT150153), specifically expressed in adult antennae, may play a more important role in the olfactory perception of *H. axyridis*. In the present study, these four antennae-specific OBPs were selected for detailed study. The development stage expression profiles of these genes were generated, and their binding characteristics to ligands were also conducted. In addition, protein structures were modeled in three dimensions, and their potential binding sites were studied by molecular docking. The results increase our comprehending of the molecular basis of olfaction of *H. axyridis* and may help to enhance their biological control effectiveness.

## Materials and Methods

### Insect Samples

*Harmonia axyridis* was obtained from Beijing Kuoye Tianyuan Biological Technology Co., Ltd., rearing in a growth chamber of the Beijing Academy of Agriculture and Forestry Sciences with the temperature of 23 ± 1°C, 16:8 h (L:D) photoperiod and 70% relative humidity. The adults and larvae were fed with aphid *Aphis craccivora* Koch (Qu et al., 2018). To determine the transcript levels of *HaxyOBP3*, *5*, *12*, and *15* under various developmental stages (eggs, first, second, third, and fourth instar, pupae, and male and female adults), samples were collected and stored at −80°C. Three biological replicates were conducted.

### Specific Expression of OBP Genes

The TRIzol reagent (Invitrogen, Carlsbad, CA, United States) was used to extract total RNA samples based on the manufacturer’s instructions. The first-strand cDNA was synthesized using the PrimeScript™ RT reagent Kit (TAKARA, Japan) following the provided protocol. The development stage expression pattern of HaxyOBPs was assessed by qRT-PCR. qRT-PCR was performed on ABI PRISM 7500 (Applied Biosystems, United States). The reaction consisted of 10 μl SYBR Premix *Ex Taq*TM II (TaKaRa, Japan), 1 μl of each primer (10 μmol L^−1^), 2 μl cDNA, 0.4 μl Rox Reference Dye II (Takara, Japan), and 5.6 μl nuclease free water. The reaction conditions were 95°C for 30 s, followed by 40 cycles of 95°C for 5 s and 60°C for 34 s. Primers of HaxyOBPs were based on Qu et al. (2021). EF1A and RPS13 genes were used as housekeeping genes (Qu et al., 2018). All samples were tested in three biological replicates. The 2^-△△CT^ method was used for relative quantification (Schmittgen and Livak, 2008). The differences in the transcript levels of HaxyOBPs in different developmental stages were compared by One-way ANOVA (SPSS 19.0, Chicago, IL, United States), followed by Tukey’s test. Heat map illustrating the log_2_ transformation of HaxyOBPs mRNA expression levels in different developmental stages.

### Expression and Purification of Recombinant OBPs

The DNA sequences that encode the HaxyOBP3, 5, 12, and 15 proteins were chemically synthesized and cloned into pET30a (+) by GenScript (Nanjing, China; Wang et al., 2020b). The positive plasmid was then transformed into BL21 (DE3) cells for the expression of recombinant proteins, and proteins induced with 0.5 mmol/L isopropyl *β*-D-1-thiogalactopyranoside (IPTG) for 4 h at 37°C (HaxyOBP3) or 16 h at 15°C (HaxyOBP5, 12, and 15). HaxyOBP5 was expressed in the supernatant. HaxyOBP3, 12, and 15 were mainly found in inclusion bodies. Inclusion bodies were denatured by 8 M urea. Recombinant proteins of HaxyOBP3, 12, and 15 were dissolved and refolded based on the reported methods (Zhang et al., 2012).

Protein purification was performed with His-Tag Purification Resin column (Genscript Biology Company, Nanjing, Jiangsu, China) and purified by gradient imidazole buffer (20, 50, 100, 250, and 500 mmol·L^−1^). The purity and size of proteins were detected by SDS-PAGE, and concentrations of proteins were measured with bicinchoninic acid (BCA) Protein Assay Kit (ThermoFisher Scientific-Life Technologies, Carlsbad, CA, United States).

### Competitive Fluorescence Binding Assay

A Cary Eclipse Fluorescence Spectrophotometer (Agilent Technologies, United States) was used to determine the results of the binding assay. *N*-phenyl-1-naphthylamine (1-NPN) for HaxyOBP15 and 4,4′-Dianilino-1,1′-binaphthyl-5,5′-disulfonic acid dipotassium salt (bis-ANS) for HaxyOBP3, 5, and 12 were chosen as the fluorescent probe. The excitation wavelength was 337 nm of 1-NPN and 295 nm of bis-ANS, and the emission spectrum was recorded between 350 and 500 nm for 1-NPN and between 300 and 550 nm for bis-ANS. The recombinant proteins prepared in Tris–HCl (50 mM, pH 7.4) was titrated with aliquots of 1 mM 1-NPN or bis-ANS to final concentrations ranging from 2 to 16 μM to measure the binding affinity. To further measure the binding affinity of ligands to HaxyOBPs, proteins and fluorescent probe at 2 μM were titrated with aliquots of 1 mM odorants. The binding constant (K_1-NPN/bis-ANS_) of 1-NPN or bis-ANS to HaxyOBPs was calculated by GraphPad Prism 5 software (GraphPad Software Inc.) with the equation K_i_ = [IC_50_]/(1 + [1-NPN]/K_1-NPN_) or [IC_50_]/(1 + [bis-ANS]/K_bis-ANS_), where [1-NPN]/[bis-ANS] is the free concentration of 1-NPN/bis-ANS, and K_1-NPN_/K_bis-ANS_ is the dissociation constant of the protein/1-NPN (bis-ANS).

### Three-Dimensional Modeling and Molecular Docking

Three-dimensional structure of HaxyOBP12 and HaxyOBP15, more than 30% homology with the OBP templates in the Protein Database,^1^ was modeled by Program MODELLER (Martí-Renom et al., 2000; Fiser et al., 2010), while HaxyOBP3 and HaxyOBP5 that had less than 30% homology were also generated using a deep residual neural network trRosetta (https://yanglab.nankai.edu.cn/trRosetta; Yang et al., 2020). Three methods, including Verify_3D, Procheck, and ERRAT were used to assess the final 3D model of HaxyOBPs protein (Laskowski et al., 1996; Webb and Sali, 2016). AutoDock Vina (version 1.1.2) was selected to analyze the binding mode between HaxyOBPs protein and compounds with the default parameters (Morris et al., 2009). The top ranked binding mode was evaluated according to the Vina docking score, and visually analyzed by PyMOL (version 1.9.0; http://www.pymol.org/).

## Results

### Developmental Stage Expression of HaxyOBPs

**Figure 1:**
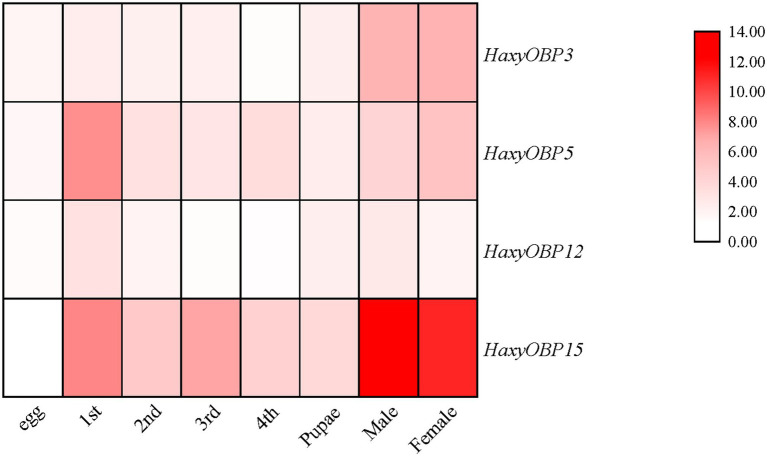
Relative expression levels of *HaxyOBP3*, *5*, *12*, and *15* genes in different developmental stages.

qRT-PCR was used to determine the expression levels of *HaxyOBP3*, *5*, *12*, and *15* in different developmental stages (Figure 1). *HaxyOBP3* and *HaxyOBP15* were both highly expressed in adults, and *HaxyOBP15* had a significantly higher expression level in this stage. Transcripts of *HaxyOBP5* were especially abundant in the first instar. In addition, *HaxyOBP12* showed similar relative transcript levels in all developmental stages.

### Expression and Purification of HaxyOBPs

**Figure 2:**
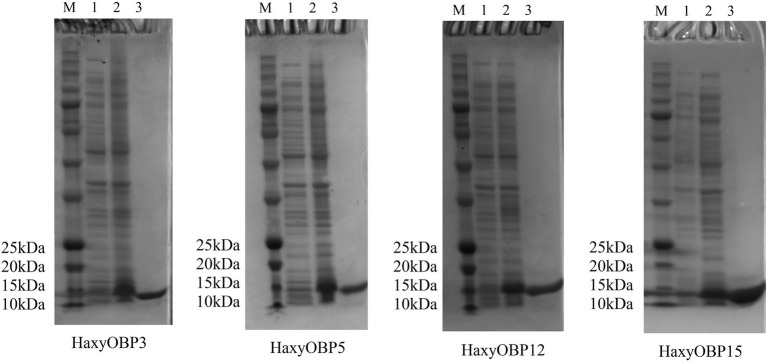
Expression and purification of HaxyOBP3, HaxyOBP5, HaxyOBP12, and HaxyOBP15. M: Marker; 1: Uninduced recombinant protein; 2: IPTG-induced recombinant protein; and 3: Purified proteins.

The recombinant proteins of HaxyOBP3, 5, 12, and 15 were successfully expressed in the *E. coli* system induced by IPTG. HaxyOBP5 was mainly detected in the supernatant, while HaxyOBP3, 12, and 15 were present in inclusion bodies (Supplementary Figure S1). Therefore, 8 mol/L urea was used to extract the protein of HaxyOBP3, 12, and 15 before the purification. Renaturation, dialysis, and ultrafiltration were then used to obtain the purified target proteins of HaxyOBP3, 12, and 15. The OBPs were purified by nickel affinity chromatography. SDS-PAGE analysis revealed the final purified proteins as a single band, a molecular weight of about 15 kDa, consistent with the predicted molecular mass (Figure 2).

### Binding Characteristics of HaxyOBPs

**Figure 3:**
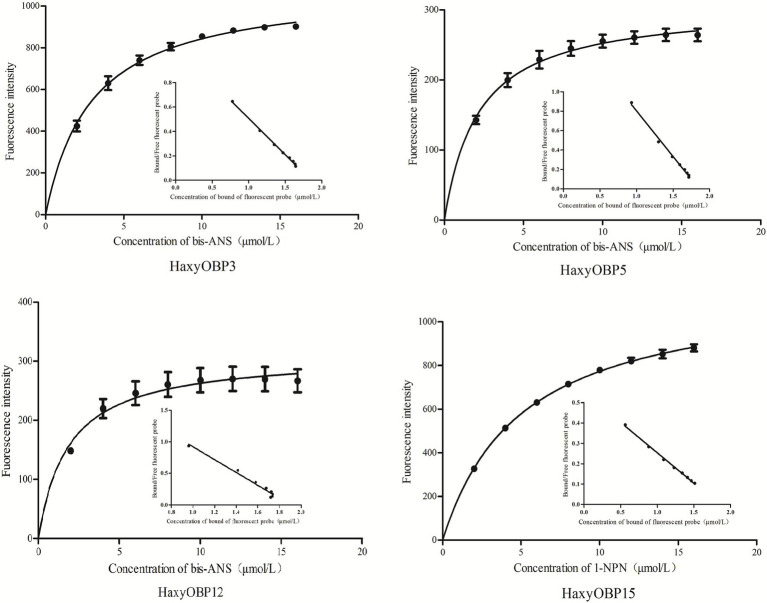
Binding curves and scatchard plots (insert) of probe to HaxyOBPs.

To determine the binding spectra of four HaxyOBPs recombinant proteins, fluorescence competitive binding assay was conducted. The binding characteristics of HaxyOBP3, 5, and 12 with fluorescent probe bis-ANS were detected by molecular fluorescence spectrometry, and the dissociation constants (K_i_ value) of HaxyOBP3, HaxyOBP5, and HaxyOBP12 were 3.07 ± 057, 2.23 ± 0.36, and 1.84 ± 0.10 μM, respectively. Using the same method, we detected the binding characteristics of HaxyOBP15 with 1-NPN, and the dissociation constant was 5.07 ± 0.31 μM (Figure 3).

**Figure 4:**
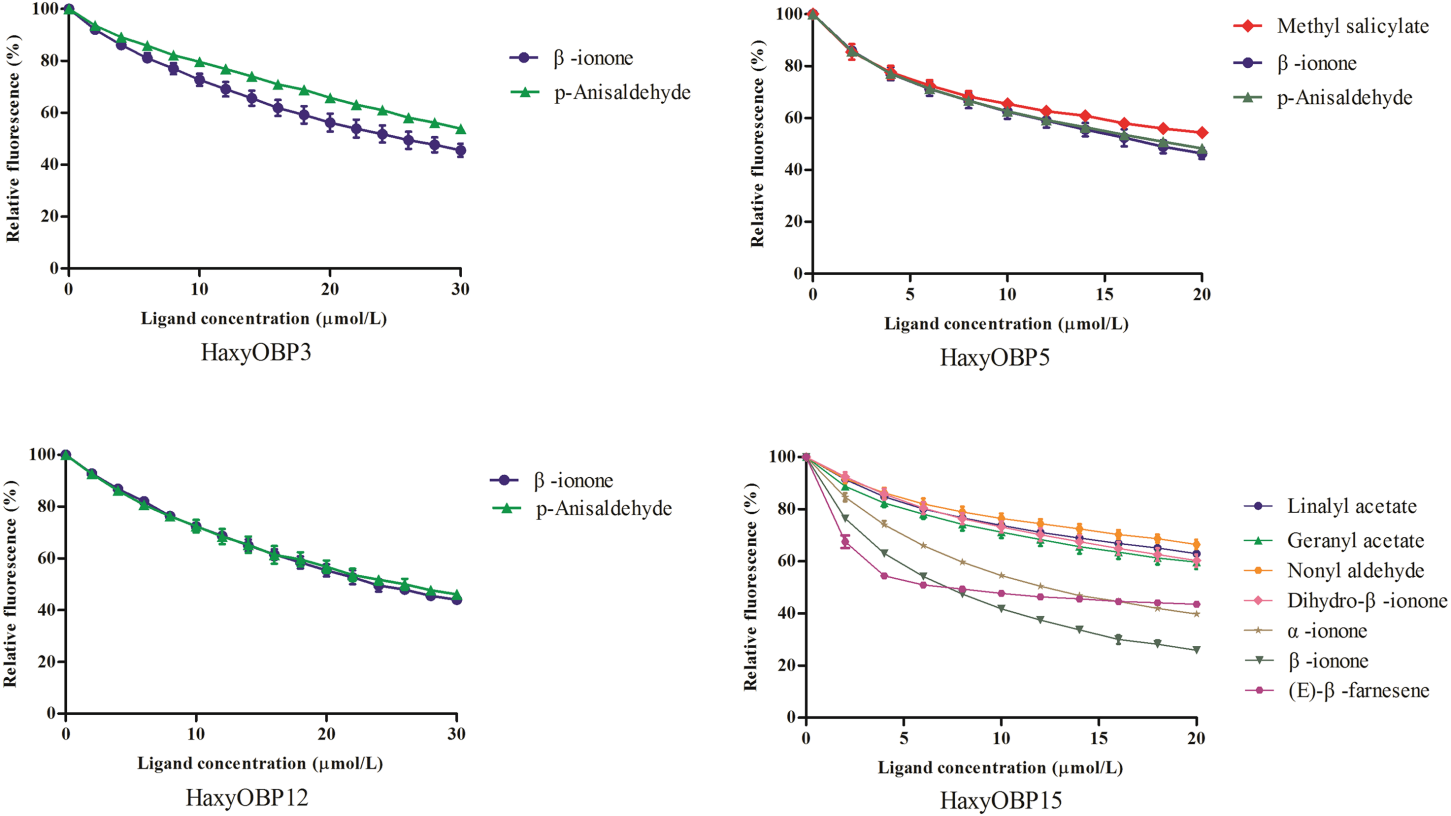
Competitive binding curves of compounds to HaxyOBP3, HaxyOBP5, HaxyOBP12, and HaxyOBP15.

Using 1-NPN or bis-ANS as a probe, 40 chemicals were used in competitive binding assay. HaxyOBP15 showed a broad binding profile with (E)-β-Farnesene, β-ionone, α-ionone, geranyl acetate, dihydro-β-ionone, nonyl aldehyde, and linalyl acetate, with the K_i_ values between 4.33 and 40.02 μM. HaxyOBP5 could bind methyl salicylate, β-ionone, and p-anisaldehyde, with the K_i_ values of 11.71, 13.45, and 18.15 μM, respectively. HaxyOBP3 and HaxyOBP12 showed narrow binding spectra and were able to only bind β-ionone and p-anisaldehyde, with the K_i_ values of 18.78 and 24.43 μM for HaxyOBP3 and 15.22 and 16.15 μM for HaxyOBP12, respectively (Figure 4; Table 1).

**Table 1:** Binding affinities of HaxyOBPs with the compounds.

Name	HaxyOBP3		HaxyOBP5		HaxyOBP12		HaxyOBP15	
	IC_50_ (μM)	K_i_ (μM)	IC_50_ (μM)	K_i_ (μM)	IC_50_ (μM)	K_i_ (μM)	IC_50_ (μM)	K_i_ (μM)
p-Anisaldehyde	34.10 ± 1.06	24.43 ± 0.37	19.93 ± 0.70	13.45 ± 0.67	25.45 ± 1.99	16.15 ± 1.21	--	--
4-Allyl-1,2-dimethoxybenzene	--	--	--	--	--	--	--	--
Nonyl aldehyde	--	--	--	--	--	--	38.49 ± 2.03	29.60 ± 1.86
α-Caryophyllene	--	--	--	--	--	--	--	--
N,N-Diethyl-m-toluamide	--	--	--	--	--	--	--	--
Cis-3-hexenyl butyate	--	--	--	--	--	--	--	--
3-Methyl-1-butanol	--	--	--	--	--	--	--	--
1-Octene	--	--	--	--	--	--	--	--
β-Caryophyllene	--	--	--	--	--	--	--	--
(−)-trans-Caryophyllene	--	--	--	--	--	--	--	--
(+)-α-Pinene	--	--	--	--	--	--	--	--
1-Octen-3-ol	--	--	--	--	--	--	--	--
Cis-3-hexen-1-ol	--	--	--	--	--	--	--	--
Phenylacetaldehyde	--	--	--	--	--	--	--	--
2-Phenylethanol	--	--	--	--	--	--	--	--
Terpinolene	--	--	--	--	--	--	--	--
Acetoin	--	--	--	--	--	--	--	--
α-Terpinene	--	--	--	--	--	--	--	--
β-Cyclocitral	--	--	--	--	--	--	--	--
β-Citronellol	--	--	--	--	--	--	--	--
(+)-2-Carene	--	--	--	--	--	--	--	--
Methyl jasmonate	--	--	--	--	--	--	--	--
α-Pinene	--	--	--	--	--	--	--	--
Geraniol	--	--	--	--	--	--	--	--
β-Ionone	25.87 ± 2.85	18.78 ± 2.14	17.77 ± 1.52	11.71 ± 0.86	24.03 ± 1.56	15.22 ± 1.06	6.99 ± 0.21	5.34 ± 0.18
P-Cymene	--	--	--	--	--	--	--	--
Tetradecane	--	--	--	--	--	--	--	--
Methyl laurate	--	--	--	--	--	--	--	--
Carvacrol	--	--	--	--	--	--	--	--
Linalool	--	--	--	--	--	--	--	--
Dihydro-β-ionone	--	--	--	--	--	--	30.89 ± 3.01	24.01 ± 2.65
α-Ionone	--	--	--	--	--	--	12.03 ± 0.56	9.21 ± 0.44
(E)-β-Farnesene	--	--	--	--	--	--	5.81 ± 0.46	4.33 ± 0.40
Methyl salicylate	--	--	26.44 ± 1.96	18.15 ± 1.33	--	--	--	--
(s)-(−)-Limonone	--	--	--	--	--	--	--	--
α-Humulene	--	--	--	--	--	--	--	--
Geranyl acetate	--	--	--	--	--	--	30.26 ± 3.58	23.38 ± 3.04
Linalyl acetate	--	--	--	--	--	--	40.02 ± 2.91	31.01 ± 2.49
Ethyl octanoate	--	--	--	--	--	--	--	--
β-Elemene	--	--	--	--	--	--	--	--

### Homology Modeling and Molecular Docking

Sequence alignments showed that HaxyOBP12 and HaxyOBP15 share 44 and 31% amino acid identities with 6JPM and 4Z45, respectively. Sequence alignments showed that HaxyOBP3 and HaxyOBP5 share 29.27 and 28.93% amino acid identities, respectively, with the templates 1C3Y and 6QQ4, less than 30.00% (Table 2). The low identity may decrease the accuracy of the predicted model. So, we used the other method, trRosetta, to predict the model of HaxyOBP3 and HaxyOBP5. The trRosetta can predict the protein more accurately for the low identity sequence. The models predicted by Homology modeling were named Mod-HaxyOBP12 and Mod-HaxyOBP15. The models predicted by trRosetta were named trR-HaxyOBP3 and trR-HaxyOBP5.

**Table 2:** Homologous templates of odorant-binding proteins (OBPs) in *Harmonia axyridis*.

Name	BLAST X match result				
	Species	PDB number of template protein	*E*-value	Identify	Score
HaxyOBP3	*Tenebrio molitor*	1C3Y	2e^−04^	29.27%	38.9
HaxyOBP5	*Drosophila melanogaster*	6QQ4	1e^−09^	28.93%	53.5
HaxyOBP12	*Chrysopa pallens*	6JPM	2e^−27^	44.00%	98.6
HaxyOBP15	*Nasonovia ribisnigri*	4Z45	3e^−09^	31.03%	52.0

For all of the predicted protein models, VERIFY3D, ERRAT, and Procheck were used to analyze the accuracy and reliability. The VERIFY3D (Supplementary Figure S2), ERRAT (Supplementary Figure S3), and Procheck (Supplementary Figure S4) showed that the models of Mod-HaxyOBP12, Mod-HaxyOBP15, trR-HaxyOBP3, and trR-HaxyOBP5 were reasonable.

**Figure 5:**
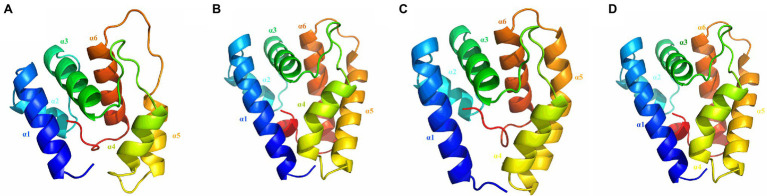
Three-dimensional structure mode of odorant binding proteins in *Harmonia axyridis* (**A**: HaxyOBP3; **B**: HaxyOBP5; **C**: HaxyOBP12; and **D**: HaxyOBP15).

The protein structures of HaxyOBP3, 5, 12, and 15 were composed of six typical α-helices, forming a hydrophobic binding cavity, which are the important features of insect OBPs (Figure 5).

According to the affinities between recombinant proteins and chemicals, we selected different numbers of ligands to study the docking conformation and binding energy with four HaxyOBPs proteins, including two ligands (β-ionone and p-anisaldehyde) for HaxyOBP3 and HaxyOBP12, three ligands (methyl salicylate, β-ionone, and p-anisaldehyde) for HaxyOBP5, and seven ligands [(E)-β-Farnesene, β-ionone, α-ionone, geranyl acetate, dihydro-β-ionone, nonyl aldehyde, and linalyl acetateone] for HaxyOBP15. The binding energy values were all negative and ranged from −5.13 to −7.31 kcal mol^−1^ (Table 3).

**Table 3:** Molecular docking analysis of ligands and its binding energy toward HaxyOBPs.

Ligand	HaxyOBP3 (kcal mol^−1^)	HaxyOBP5 (kcal mol^−1^)	HaxyOBP12 (kcal mol^−1^)	HaxyOBP15 (kcal mol^−1^)
β-Ionone	−6.11	−5.95	−5.60	−6.13
p-Anisaldehyde	−5.89	−5.84	−5.86	--
Methyl salicylate	--	−5.13	--	--
Dihydro-β-ionone	--	--	--	−6.19
α-Ionone	--	--	--	−5.83
(E)-β-Farnesene	--	--	--	−7.31
Geranyl acetate	--	--	--	−6.94
Linalyl acetate	--	--	--	−6.09
Nonyl aldehyde	--	--	--	−6.32

**Figure 6:**
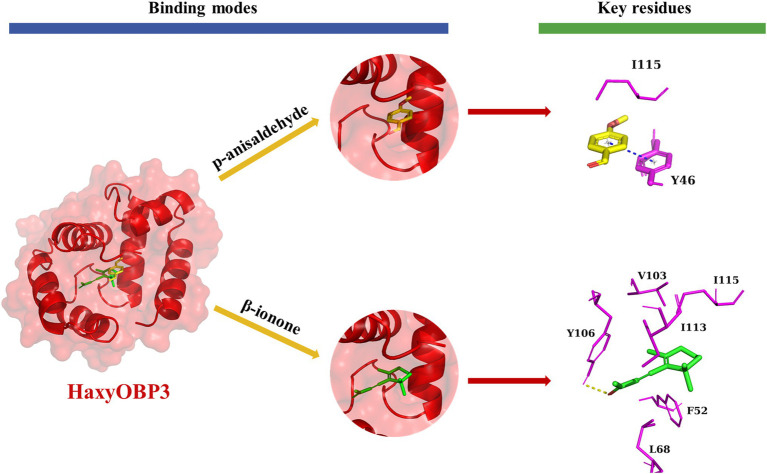
Binding pattern of HaxyOBP3 with p-anisaldehyde and β-ionone.

For HaxyOBP3, p-anisaldehyde bound the protein with Y46 and I115 and formed a “π-π” interaction with Y46. β-ionone bound the protein with F52, L68, V103, I113, and I115. The ligand formed hydrogen bond interactions with Y106. I115 is a common key residue for two ligands (Figure 6).

**Figure 7:**
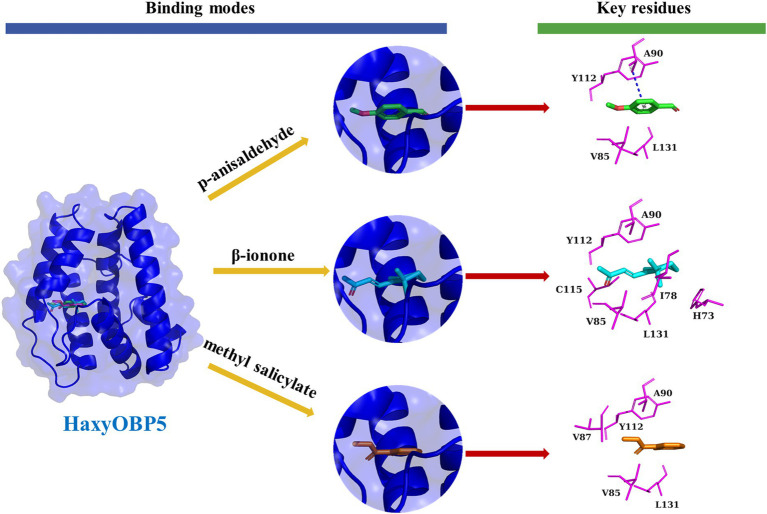
Binding pattern of HaxyOBP5 with p-anisaldehyde, β-ionone, and methyl salicylate.

For HaxyOBP5, seven residues, including H73, I78, V85, A90, Y112, C115, and L131, were critical for binding affinity to β-ionone based on hydrophobic interactions. P-anisaldehyde formed a “π-π” interaction with Y112 of HaxyOBP5 and formed hydrophobic interactions with V85, A90, and L131. Methyl salicylate formed hydrogen bond interactions with L131 of HaxyOBP5 and hydrophobic interactions with V85, V87, A90, and Y112 (Figure 7).

**Figure 8:**
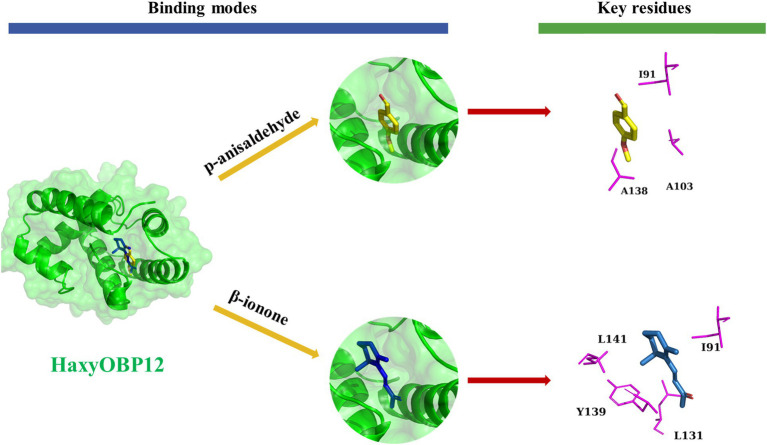
Binding pattern of HaxyOBP12 with p-anisaldehyde and β-ionone.

Hydrophobic interactions were the important linkages between HaxyOBP12 and β-ionone and p-anisaldehyde. Three residues, including I91, A103, and A138, were critical for binding affinity to p-anisaldehyde. Four residues, including I91, L131, Y139, and L141, were critical for binding affinity to β-ionone (Figure 8).

**Figure 9:**
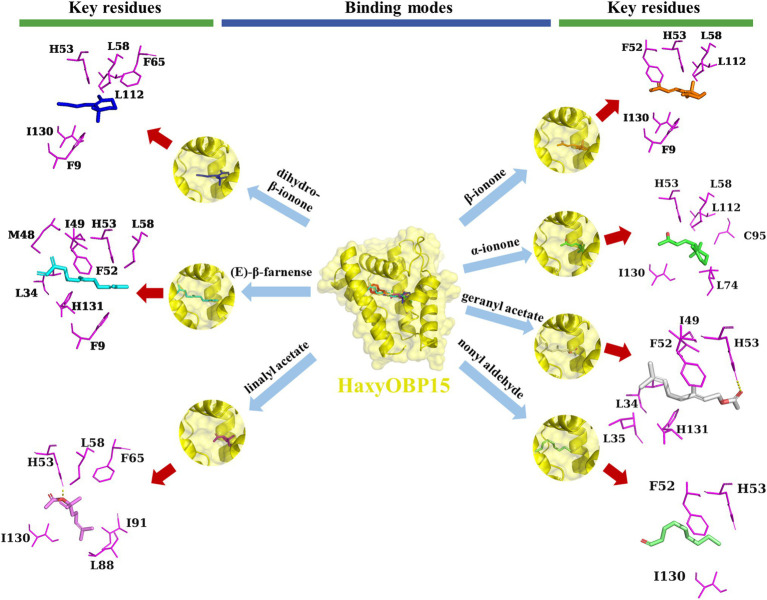
Binding pattern of HaxyOBP15 with β-ionone, dihydro-β-ionone, α-ionone, EBF, geranyl acetate, linalyl acetate, and nonyl aldehyde.

For HaxyOBP15, hydrophobic interactions were the important linkages between HaxyOBP15 and β-ionone, dihydro-β-ionone, and α-ionone. Three residues, including H53, L58, and I130, appeared to be involved in the binding affinity to the three substances. (E)-β-Farnesene and HaxyOBP15 also have hydrophobic interactions, mediated by F9, L34, M48, I49, F52, H53, and L58. A hydrogen bonding interaction existed between HaxyOBP15 and geranyl acetate and linalyl acetate, with the key residue H53, and there were some hydrophobic residues involved in the interactions (Figure 9).

## Discussion

Odorant-binding proteins are the front-line environmental odorant sensors, playing an essential role in insect behavior (Pelosi et al., 2006). Temporal and spatial expression patterns of OBPs in insects are interrelated with their specific physiological functions (Song et al., 2014; Sun et al., 2016; Zhang et al., 2017b). The transcripts of *HaxyOBP3*, *5*, *12*, and *15* were mainly restricted to adult antennae in our previous study (Qu et al., 2021), implying a role of these proteins in olfactory chemoreception. Clarifying the expression characteristics of insects’ OBPs at different developmental stages can also help to understand their functions in olfactory recognition (Ju et al., 2014). In this study, qRT-PCR indicated that *HaxyOBP3*, *5*, *12*, and *15* had different transcript levels during the different developmental stages of *H. axyridis*. *HaxyOBP5* and *HaxyOBP12* were abundant in the larval stage, indicating their connection to larval biological characteristics of *H. axyridis*. However, *HaxyOBP3* and *HaxyOBP15* were both highly expressed in adult stage, and the expression level of *HaxyOBP15* was significantly higher in this stage, indicating they might be involved in adult-specific behaviors.

The results of the fluorescence binding assay also showed that HaxyOBP15 had a broader ligand-binding affinity, and it could bind seven substances including (E)-β-Farnesene, β-ionone, α-ionone, geranyl acetate, dihydro-β-ionone, nonyl aldehyde, and linalyl acetate, comparing with HaxyOBP3, 5, 12. These results were consistent with the fact that *HaxyOBP15* gene showed significantly higher expression level in adult stage, indicating that *HaxyOBP15* played a key role in olfactory communication of adult *H. axyridis*. *Harmonia axyridis* is an important natural enemy in many crops (Koch, 2003; Pervez and Omkar, 2006). Plant volatiles and sex pheromone are essential signal chemicals in pest-crop-natural enemy interactions (Li et al., 2018). Compared with larvae, adults of *H. axyridis* have a wider range of activities due to their ability to fly, and they need to recognize more odorants, so as to detect food sources or find suitable oviposition sites.

Homology modeling and molecular docking were used to further study the specific binding characteristics of OBPs. Classic OBPs usually has six α-helical domains, and fold together to form a compact pocket for combing odors (Pelosi et al., 2014). The present study showed that the predicted 3D structures of the four HaxyOBPs were consistent with those of classic OBPs, having six α-helical domains. Ligands are usually bound in a hydrophobic cavity of insect OBPs (Wogulis et al., 2006; Northey et al., 2016). In this study, molecular docking results showed that HaxyOBP15 broadly bound with more substances, suggesting that HaxyOBP15 may have adapted to binding to substances with different shapes and sizes. Some residues of HaxyOBP15 may specifically interact with functional groups of substances. For instance, HaxyOBP15 possess a key amino acid residue, H53, which appears to be involved in the recognition of a broad range of substances.

Among the 40 candidate compounds, nine compounds, including β-ionone, α-ionone, dihydro-β-ionone, geranyl acetate, nonanal, linalyl acetate, EBF, p-anisaldehyde, and methyl salicylate, bound to four HaxyOBPs according to fluorescence binding assay and molecular docking. β-ionone as a fragrance compound, existing in the flowers and fruits of many plants (Beekwilder et al., 2014; Fu et al., 2019; Guarino et al., 2021) and having a strong repellent effect on flea beetles, spider mites, and whiteflies (Caceres et al., 2016), could bind with the four HaxyOBPs proteins. Moreover, HaxyOBP15 could also bind with α-ionone and dihydro-β-ionone, the analogs of β-ionone. For HaxyOBP15, hydrophobic interactions played a key role in the binding of HaxyOBP15 to three substances based on molecular docking. Three substances have similar chemical structures, and also commonly exist in plant volatiles, playing an important role in interactions between plants and insects (Li et al., 2019a). For example, Dihydro-β-ionone is attractive to the crucifer flea beetle (Caceres et al., 2016). The bouquet of *Philodendron adamantinum* is mainly composed of dihydro-β-ionone, which can attract the beetle *Erioscelis emarginata* and promote the pollination process (Pereira et al., 2014). The α-ionone was widely used as a male attractant for *Bractocera latifrons* (Nishida et al., 2009).

HaxyOBP15 could also bind geranyl acetate, nonanal, and linalyl acetate, and H53 was the key residue between HaxyOBP15 and these three substances by molecular docking. Geranyl acetate is similar to (E)-ß-farnesene (EBF) in structure, but the polarity and hydrophilicity of two compounds are different. Geranyl acetate is an ester, and EBF is a hydrocarbon. Previous studies reported that geranyl acetate is a strong activator of OR5 of aphids and also has binding affinity to OBP3 and OBP7, demonstrating features shared by several other behaviorally active repellents (Zhang et al., 2017a). Linalyl acetate is a monoterpene ester, which can be isolated from essential oils of *Chrysactinia mexicana*, *Lavandula angustifolia*, and *Thymus leptophyllus* (Jan et al., 2016). In addition, the common volatile compound nonanal can attract female *Grapholitha molesta* in Y-tube experiment and is a critical volatile of tobacco for attracting female *Helicoverpa assulta* (Lu and Qiao, 2020; Wang et al., 2020a).

More importantly, fluorescence binding assay showed that HaxyOBP15 exhibited the strongest binding affinity with EBF. Molecular docking results also revealed that HaxyOBP15 and EBF displayed the strongest binding activity, having hydrophobic interactions mediated by F9, L34, M48, I49, F52, H53, and L58, with the lowest binding energy values. EBF, as main active component of aphid alarm pheromone (Bowers et al., 1977), can cause aphids to kick, stop feeding, and disperse from feeding site (Pickett et al., 1992), and mediates the winged morphs’s production (Kunert et al., 2005). Many aphid predators, such as hoverflies (Harmel et al., 2007), ground beetles (Kielty et al., 1996), and lady beetles (Nakamuta, 1991; Verheggen et al., 2007; Liu et al., 2014), utilize the aphid alarm pheromone EBF as a foraging cue. OBP3, 7, and 9 are associated with EBF perception in aphids (Qiao et al., 2009; Sun et al., 2011; Northey et al., 2016; Fan et al., 2017; Qin et al., 2020; Wang et al., 2021). The OBP1 of *Chrysoperla sinica* was able to bind EBF (Li et al., 2018), and the OBP10 of *Chrysopa pallens* mediated the perception of EBF (Li et al., 2017).

Although HaxyOBP3, 5, and 12 had a relatively narrow binding spectrum, comparing with HaxyOBP15, but they all bound to p-anisaldehyde. P-anisaldehyde is a naturally occurring fragrant phenolic compound that exists in anise, cumin, fennel, garlic, and other plant species (Boulogne et al., 2012). P-anisaldehyde is also a chemical communication substance of many insects (El-Sayed et al., 2008; Mainali and Lim, 2011; Thoming et al., 2020). For example, p-anisaldehyde can attract *Frankliniella occidentalis* and *Thrips tabaci* (Hollister et al., 1995; Koschier et al., 2000), and is an effective attractant for adults of *Anthrenus verbasci* (Imai et al., 2002). However, p-anisaldehyde has a repellent effect on some species, including *Amblyomma americanum* (Showler and Harlien, 2018) and *Musca domestica* (Showler and Harlien, 2019). In addition, HaxyOBP5 could bind with methyl salicylate (MeSA), a herbivore-induced plant volatile that is attractive to many predators such as ladybeetles (James, 2003a; Zhu and Park, 2005; Salamanca et al., 2017), lacewings (James, 2003b), hoverflies (Mallinger et al., 2011), mites (de Boer and Dicke, 2005), bugs (James, 2005), and aphid parasitoids (Gordon et al., 2013; Martini et al., 2014). In addition, MeSA has repellent effect on several aphid species (Ninkovic et al., 2003; Wang et al., 2019).

In summary, the expression levels of the four HaxyOBPs genes showed different in development stages, and HaxyOBP15 was significantly higher expressed in the adult of *H. axyridis* based on the results of qRT-PCR. Ligand binding assays and molecular docking demonstrated HaxyOBP15 exhibited high specificity for more substances, comparing with HaxyOBP3, 5, and 12, suggesting HaxyOBP15 may play the most prominent role in the olfactory chemoreception of *H. axyridis.* These results can provide insight into the mechanism of olfactory communication of *H. axyridis* and enhance the biological control effectiveness of *H. axyridis*.

## Data Availability Statement

The original contributions presented in the study are included in the article/Supplementary Material, further inquiries can be directed to the corresponding authors.

## Author Contributions

CQ and CL conceived and designed the experiments. CQ performed the experiments. CQ and F-qL analyzed the data. Z-kY and CQ provided the homology modeling and molecular docking and wrote the initial manuscript. CQ and H-pZ prepared the figures. CL, X-lY, and SW edited and reviewed the manuscript. All authors accepted the final version of the manuscript.

## Funding

This work was supported by the China Agriculture Research System of MOF and MARA (CARS-24-C-03); the National Key Research and Development Program of China (2017YFD0200400); and the Shandong Province Modern Agricultural Technology System Peanut Innovation Team, China (SDAIT-04-08).

## Conflict of Interest

The authors declare that the research was conducted in the absence of any commercial or financial relationships that could be construed as a potential conflict of interest.

## Publisher’s Note

All claims expressed in this article are solely those of the authors and do not necessarily represent those of their affiliated organizations, or those of the publisher, the editors and the reviewers. Any product that may be evaluated in this article, or claim that may be made by its manufacturer, is not guaranteed or endorsed by the publisher.

## Footnotes

^1^ http://www.rcsb.org
