# Supplementary figures and images for: Binding Affinity Characterization of Four Antennae-Enriched Odorant-Binding Proteins From Harmonia axyridis (Coleoptera: Coccinellidae)
Source: Front Physiol. 2022 Mar 8;13:829766. doi: 10.3389/fphys.2022.829766 (PMC8957989; doi:10.3389/fphys.2022.829766)

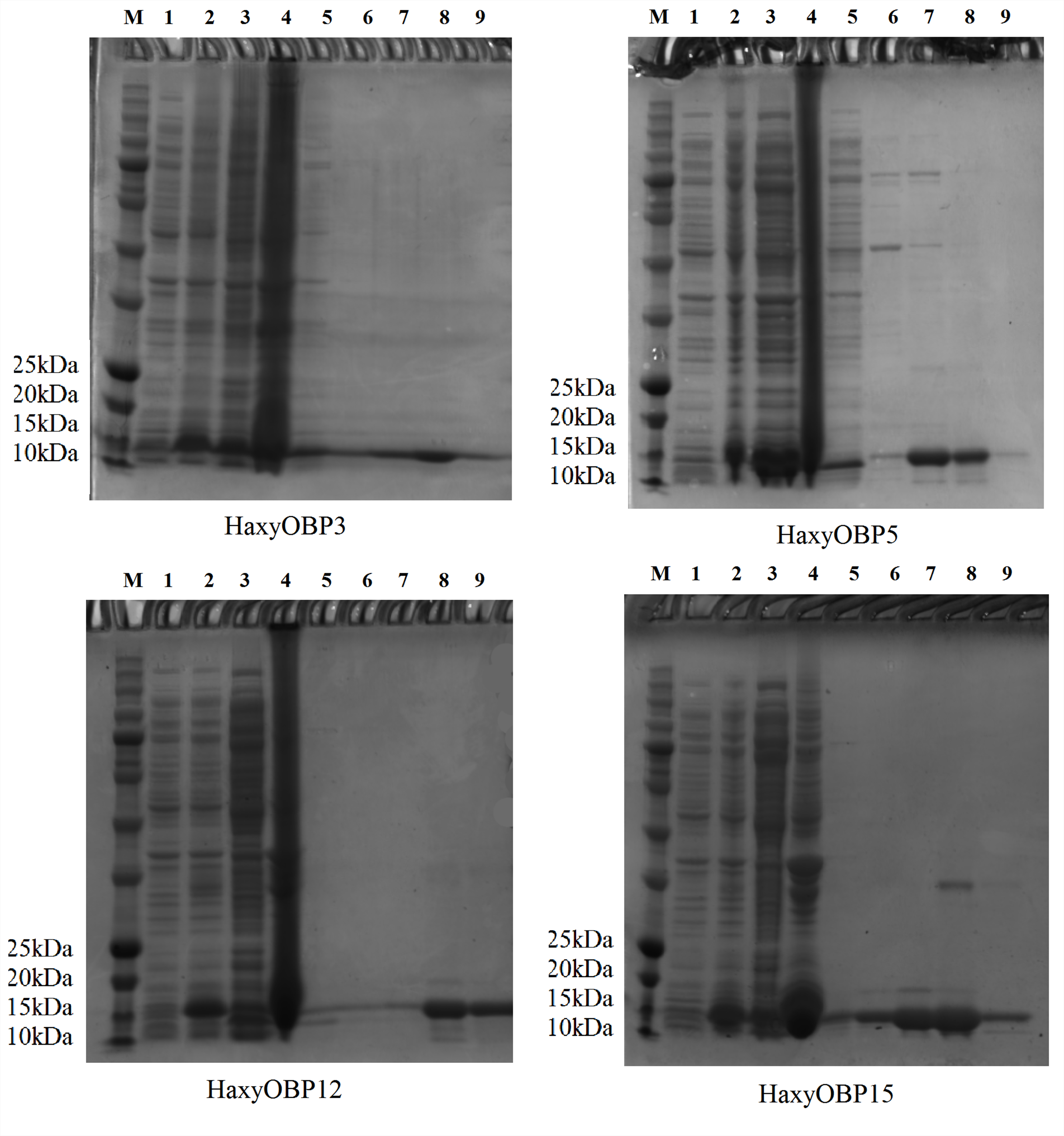

Supplement: Supplementary file 1 [file Image_1.TIF]

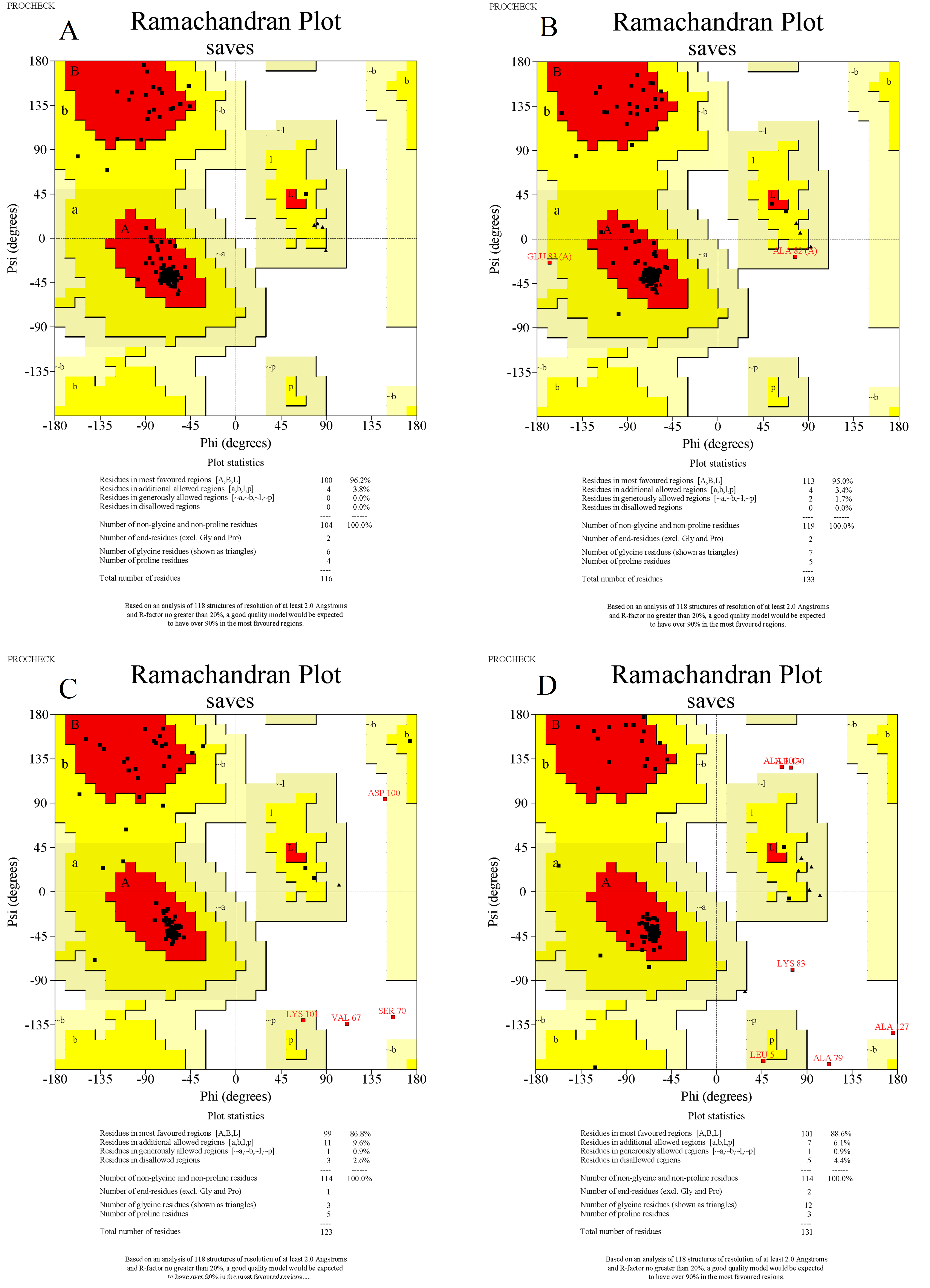

Supplement: Supplementary file 2 [file Image_2.TIF]

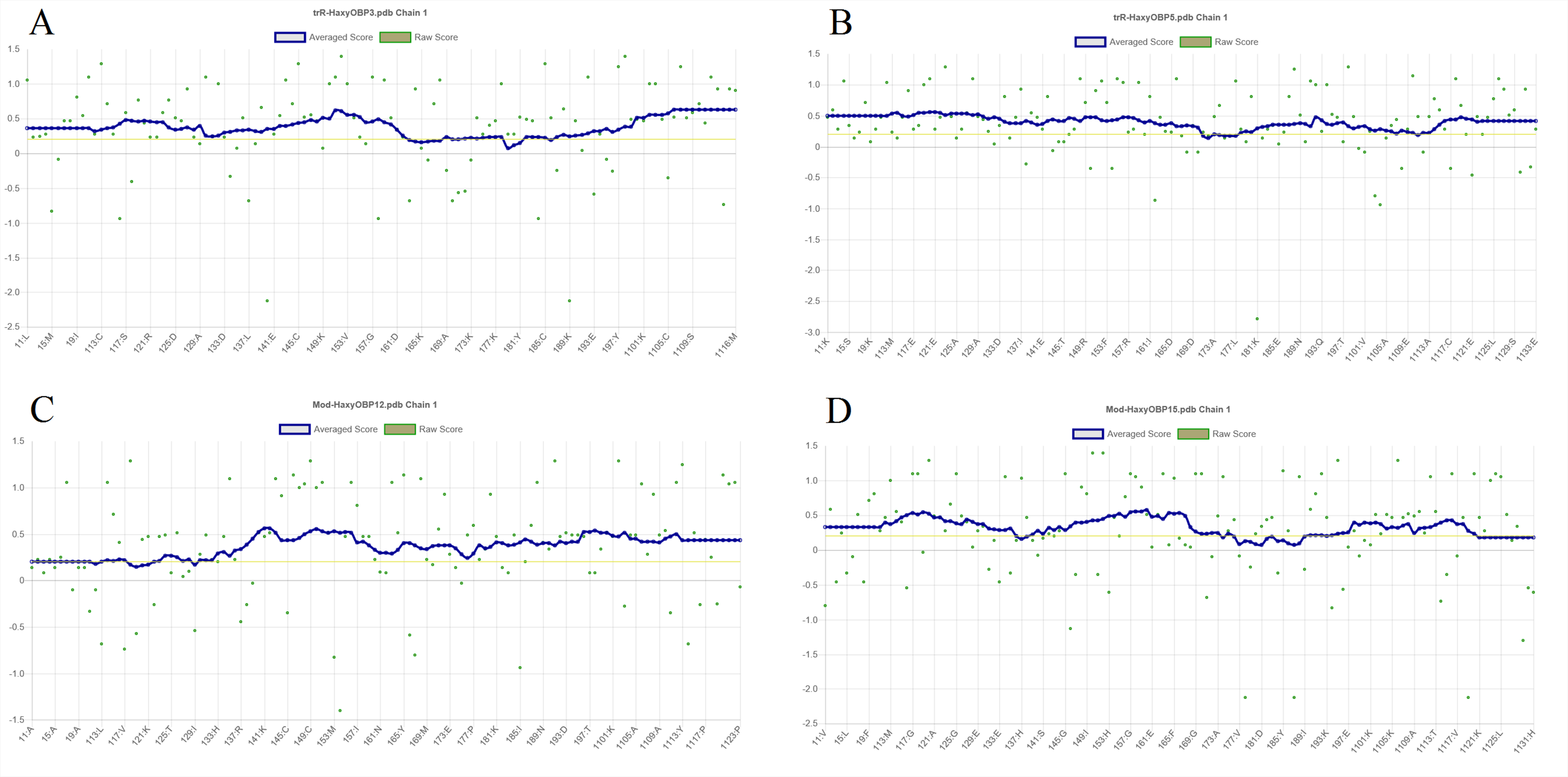

Supplement: Supplementary file 3 [file Image_3.TIF]

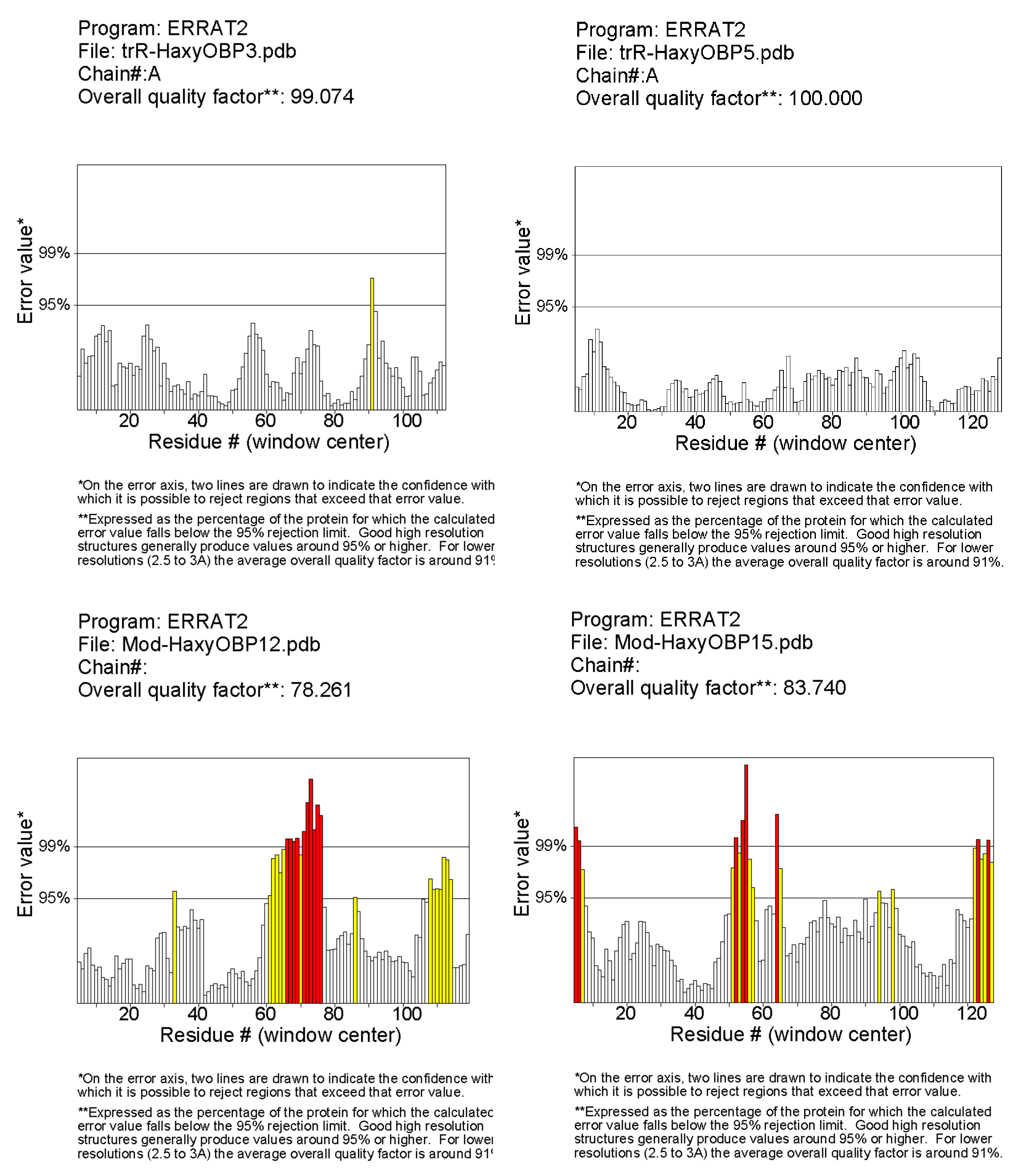

Supplement: Supplementary file 4 [file Image_4.TIF]
