# Supplementary material for: Binding Affinity Characterization of Four Antennae-Enriched Odorant-Binding Proteins From Harmonia axyridis (Coleoptera: Coccinellidae)
Source: Front Physiol. 2022 Mar 8;13:829766. doi: 10.3389/fphys.2022.829766 (PMC8957989; doi:10.3389/fphys.2022.829766)
Supplement: Supplementary file 5 [file Table_1.DOC]

Table S1 List of ligands for fluorescent binding assay

| Company | Content | CAS Number | Name |
| --- | --- | --- | --- |
| J&K Scientific | 99% | 123-11-5 | p-Anisaldehyde |
| TCI | 98% | 93-15-2 | 4-Allyl-1,2-dimethoxybenzene |
| J&K Scientific | 95% | 124-19-6 | Nonyl aldehyde |
| TCI | 93% | 6753-98-6 | Alpha-caryophyllene |
| J&K Scientific | 95% | 134-62-3 | N,N-Diethyl-m-toluamide |
| Macklin | 98% | 16491-36-4 | Cis-3-hexenyl butyate |
| J&K Scientific | 99% | 123-51-3 | 3-Methyl-1-butanol |
| J&K Scientific | 99% | 111-66-0 | 1-Octene |
| J&K Scientific | 90% | 87-44-5 | β-Caryophyllene |
| Sigma | 98.5% | 87-44-5 | (-)-trans-Caryophyllene |
| Sigma | 99% | 7785-70-8 | (+)-α-Pinene |
| J&K Scientific | 98% | 3391-86-4 | 1-Octen-3-ol |
| J&K Scientific | 98% | 928-96-1 | Cis-3-hexen-1-ol |
| Aladdin | 95% | 122-78-1 | Phenylacetaldehyde |
| Aladdin | 99% | 60-12-8 | 2-Phenylethanol |
| J&K Scientific | 95% | 586-62-9 | Terpinolene |
| TCI | 99% | 513-86-0 | Acetoin |
| Sigma | 95% | 99-86-5 | α-Terpinene |
| Aladdin | 95% | 432-25-7 | β-Cyclocitral |
| Sigma | 95% | 106-22-9 | β-Citronellol |
| Sigma | 97% | 4497-92-1 | (+)-2-Carene |
| Sigma | 95% | 39924-52-2 | Methyl jasmonate |
| Sigma | 98% | 80-56-8 | α-Pinene |
| Sigma | 97% | 106-24-1 | Geraniol |
| Sigma | 96% | 14901-07-6 | β-Ionone |
| Sigma | 99% | 99-87-6 | P-cymene |
| Sigma | 99% | 629-59-4 | Tetradecane |
| Aladdin | 99% | 111-82-0 | Methyl laurate |
| J&K Scientific | 98% | 499-75-2 | Carvacrol |
| Sigma | 97% | 78-70-6 | Linalool |
| Sigma | 90% | 17283-81-7 | Dihydro-β-ionone |
| Aladdin | 90% | 127-41-3 | α-Ionone |
| Macklin | 95% | 18794-84-8 | (E)-β-Farnesene |
| Sigma | 99% | 119-36-8 | Methyl salicylate |
| Sigma | 96% | 5989-54-8 | (s)-(-)- Limonene |
| Sigma | 96% | 6753-98-6 | α-Humulene |
| Sigma | 97% | 105-87-3 | Geranyl acetate |
| J&K Scientific | 95% | 115-95-7 | Linalyl acetate |
| Sigma | 99% | 106-32-1 | Ethyl octanoate |
| Shanghai yuanye Bio-Technology | 97% | 515-13-9 | β-Elemene |

Fig. S1 Expression and purification of HaxyOBP3, HaxyOBP5, HaxyOBP12, HaxyOBP15

M: Marker; 1: Uninduced recombinant protein; 2: IPTG induced recombinant protein; 3: Supernatant of recombinant protein; 4: Inclusion of recombinant protein; 5: Washed protein with 20 mM imidazole; 6: Washed protein with 50 mM imidazole; 7: Washed protein with 100 mM imidazole; 7: Washed protein with 250 mM imidazole; 8: Washed protein with 500 mM imidazole

Fig. S2 Ramachandran plots of odorant binding proteins in *Harmonia axyridis*

Red: The best region, including A, B and L areas; bright yellow: the appropriate region, including a, b, l, p areas; pale yellow: the barely permitted region, including ~a, ~b, ~l, ~p areas; white: the disallowed region.

(A: HaxyOBP3; B: HaxyOBP5; C: HaxyOBP12; D: HaxyOBP15)

Fig. S3 Verify_3D scores for *Harmonia axyridis* odorant bidning proteins models

(A: HaxyOBP3; B: HaxyOBP5; C: HaxyOBP12; D: HaxyOBP15)

Fig. S4 Error values of *Harmonia axyridis* odorant binding proteins models structure residues evaluated by using ERKAT
